# The flexible, the stereotyped and the in‐between: putting together the combinatory tool use origins hypothesis

**DOI:** 10.1002/brv.70123

**Published:** 2025-12-30

**Authors:** Jennifer A. D. Colbourne, Alice M. I. Auersperg

**Affiliations:** ^1^ Messerli Research Institute, University of Veterinary Medicine Vienna Veterinärplatz 1 1210 Vienna Austria

**Keywords:** tool use, tooling, object manipulation, object combinations, object play, extractive foraging, physical cognition

## Abstract

Tool use research has long made the distinction between tool using that is considered learned and flexible, and that which appears to be instinctive and stereotyped. However, animals with an inherited tool use specialisation can exhibit flexibility, while tool use that is spontaneously innovated can be limited in its expression and facilitated by predispositions for ecological specialisations. Furthermore, recent evidence does not support the proposed division of flexible tool use along primate–bird taxonomic lines. Instead, we hypothesise that tool use is a more complementary phenomenon than previously believed, in that the intrinsic motivation for combinatory object manipulation underlies the onset of all allocentric tool use, resulting in a spectrum. What influences the initial tool use that does emerge is the form of a species' object combinations, which is itself influenced by ecological niche. Therefore, an opportunistic extractive forager will likely develop more diverse forms of tool use than a specialist.

## I. INTRODUCTION

In his landmark book *Animal Behaviour*, C. Lloyd Morgan (1900) described the digger wasp (*Ammophila* spp.) pounding its burrow closed with a stone: ‘Here’, he wrote, ‘we have intelligent behaviour rising to a level to which some would apply the term rational’ (p. 128). However, as tool use research in non‐human animals progressed, it became clear that there was a qualitative difference between the uniform, species‐wide pounding behaviour of the digger wasp and, for instance, the flexible use of a tool set to open up and ‘fish’ inside termite mounds by the chimpanzee (*Pan troglodytes*; Suzuki, Kuroda & Nishihara, 1995). Historically, early tool use researchers largely applied the (now rejected) nature–nurture divide onto tool use observations, suggesting that tool‐using animals either operated on pure instinct, or were capable of learning tool use after an accidental discovery, observational learning, or even insight (e.g. Alcock, 1972; Hall, 1963; van Lawick‐Goodall, 1971). These two types of tool use were considered so disparate that Hall (1963) argued that the only reason they were implied to be a single phenomenon was because they both share a superficial similarity to human tool use.

However, although the distinction between learned and instinctive tool use would linger, this divide shifted in favour of categorising tool‐using species according to the underlying variation of their tool use behaviour. That is, either a species' tool use is limited to a certain context and does not vary between individuals, as it requires little to no learning, (‘context specific’, ‘stereotyped’, ‘specialised’), or there is accommodation to different situations and differences between individuals/groups [‘intelligent’, ‘flexible’, ‘creative’ (e.g. Call, 2013; Hunt, Gray & Taylor, 2013; Parker & Gibson, 1977)]. We believe that treating tool use in a dichotomous fashion has been counterproductive for making comparisons between tool‐using animals and has hindered the development of a more comprehensive understanding concerning the onset of tool use. We hypothesise that there is a common trait underlying the origination of all tool behaviour in its initial expression, whether in the digger wasp or the chimpanzee, which interacts with species' underlying cognitive capacities and ecological history, resulting in a rich spectrum of tool use across the animal kingdom: the intrinsic motivation to combine objects.

## II. THE FLEXIBLE AND THE STEREOTYPED

### (1) Existing explanations for the evolution of stereotyped tool use

There has not been much debate about the origin of stereotyped, context‐specific tool use. It is typically identified by its ubiquity within species, unvaried development in the absence of social contact, lack of individual variation, and limitation to specific contexts (Hunt *et al*., 2013). It is, essentially, tool use that becomes a species‐typical action pattern. Due to its uniform expression, this type of tool use has been described as a possible behavioural adaptation standing in for a more costly morphological adaptation (Alcock, 1972; Hall, 1963; Parker & Gibson, 1977; van Lawick‐Goodall, 1971).

Alcock (1972) made the case that such tool use derived from pre‐existing behaviour patterns, in which an object was accidentally used as a tool in a specific situation. For example, he suggested that a woodpecker finch (*Camarhynchus pallidus*) that inserted its beak into a crevice when it happened to be carrying a twig, due to nesting behaviour, could result in an insect accidentally being dislodged so that the bird was rewarded with food, thereby reinforcing its repetition. Hunt *et al*. (2013), however, argued that this process required phenotypic change in order to evolve, and that only a few pre‐existing behaviour patterns could become tool use in this way. Nest building in woodpecker finches, they argued, was not closely associated enough with the necessary context (foraging) for such phenotypic evolution. Rather, the pre‐existing behaviour needed to be very similar to, and in the same context as, the tool‐using behaviour. They proposed that this can only occur when behaviours that already incorporate objects (‘object‐related mechanical actions’) are in the appropriate context to become ‘dynamic mechanical interactions’ with those objects (i.e. tool use, *sensu* St Amant & Horton, 2008) (Hunt *et al*., 2013). Thus, the digger wasp's pre‐existing object‐related behaviour of placing stones on a burrow may become the dynamic use of a stone as a tool to compact the burrow (Hunt *et al*., 2013). As the phenotypic evolution of this type of tool use relies on both the pre‐existence of appropriate object‐related actions and sufficient fitness benefits (‘utility’), it occurs only relatively rarely (Hunt *et al*., 2013).

Explanations for the pre‐conditions of flexible tool use have been far more varied. From the Piagetian perspective, a level of sensorimotor development for ‘experimentation’ (stage 5) and ‘insight’ (stage 6) needs to be reached (Chevalier‐Skolnikoff, 1989) or at least the ability to apply complex object manipulation schemata to different situations, which is a characteristic of tertiary sensorimotor intelligence (Parker & Gibson, 1977). Call (2013) has proposed three ingredients for flexible tool use, which include information hoarding, information recombination, and a propensity for object manipulation (that is, the readiness with which a species manipulates objects). On the other hand, Hunt *et al*. (2013) argued that fine object manipulative ability, processing capacity and conceptual learning (in its most basic form, that a tool can affect a target) constrain the development of flexible tool use. In the human literature, there is ongoing debate concerning the role and relative importance of manipulation knowledge (how to use a tool) and mechanical knowledge (reasoning about physical effects) in using tools (for a review, see Osiurak & Badets, 2016). Although compelling cases can be made for any number of components deemed requisite to flexible tool use, the issue remains that none is unique only to tool users, nor do their presence in any species predict its emergence with any certainty. All of these components are undoubtedly important to general technical problem solving, but they are nevertheless not specific to the phenomenon of tool use *per se*.

### (2) Suggested requirements for flexible tool use

#### (a) Tool use flexibility and the brain

Underlying each of these explanations is the assumption that a certain cognitive threshold is necessary for flexible tool use to emerge. Certainly, there is support for the association between tool‐using taxa and brain areas linked with higher order cognition. In primates, the absolute and relative size of the ‘executive brain’ (neocortex and striatum) is positively correlated to several measures of behavioural flexibility, including tool use, and brain size more generally has a strong relationship with tool use innovation (Navarrete *et al*., 2016; Reader & Laland, 2002). In a principal component and factor analysis of primates, Reader, Hager & Laland (2011) showed that tool use loads onto a single cognitive component, along with innovation, social learning, extractive foraging and tactical deception, which they described as a single general intelligence factor (*g*). This primate *g* factor further was found to correlate with several measures of neocortex/executive brain volume, and to predict performance on laboratory tests of cognition (Reader *et al*., 2011).

Across avian species, there is substantial evidence for a relationship between innovation and brain size/neuron number, especially technical innovation, which includes tool use (Audet *et al*., 2024; Lefebvre *et al*., 1997, 1998; Overington *et al*., 2009; Sol *et al*., 2005, 2022). Specifically, compared to non‐tool users (including species engaging in borderline/proto‐tool use), tool‐using bird species have a greater average brain size and a relatively larger nidopallium, a telencephalic structure considered an equivalent to the mammalian neocortex (Lefebvre, Nicolakakis & Boire, 2002). These species also have greater folding of the cerebellum, a brain area involved in movement coordination, motor learning, sensory integration, and cognitive processing (Iwaniuk, Lefebvre & Wylie, 2009). Lefebvre and colleagues have argued that such parallel trends in the brains of primates and birds are the result of convergent evolution (Lefebvre, Reader & Sol, 2004; Lefebvre & Sol, 2008).

Interestingly, neurocognitive research has not made the distinction between ‘stereotyped’ and more flexible kinds of tool use. It has been assumed more generally that smaller‐brained taxa, like insects and fish, are only capable of the former (e.g. Alcock, 1972; Hunt *et al*., 2013). However, more recent evidence has shown that various insect species use objects with a remarkable degree of flexibility, although there is still little research that has been conducted with tool/object‐using species, particularly with regard to cognition (Wen *et al*., 2024). In teleost fishes, no direct association has been found between brain size and tool/object use, although the wrasse family, which includes tool users, have relatively larger brains than expected (although not the tool‐using species within this family, however data are limited; Brown, 2012). In the absence of more research, it is still premature to make a hard divide, as suggested by Alcock (1972), between the tool use of insects and fish from that of primates and birds.

#### (b) *Bird route* versus *primate route*

Alcock (1972) is not the only scientist who proposed a separation in tool use quality across taxonomic lines, as others have taken the position that tool use in primates and birds is fundamentally different. Hansell (2000) argued that, whereas tool use in hominids and some primates may indicate advanced cognitive abilities, tool use in birds is not more manipulatively complex or deserving of special status than nest building. It was contended that tool use, like other construction behaviours, can generally be explained by underlying genetic mechanisms and possibly by some learning through experience (Hansell, 2000; Hansell & Ruxton, 2008). Relatedly, early in tool use research, it was suggested that tool use in birds originated through nest building (Alcock, 1972; Hall, 1963) and there are still claims that the two phenomena are closely related (Arbib *et al*., 2023; Healy, Tello‐Ramos & Hébert, 2023). However, although many birds use sticks to build nests, very few develop tool use, and those that do, do so in a foraging context removed from nest building (Hunt *et al*., 2013). Additionally, avian species that do not have nest building ancestry have emerged as tool users, such as the Goffin's cockatoo, also known as the Tanimbar corella (*Cacatua goffiniana*) (Auersperg *et al*., 2012; Mioduszewska *et al*., 2019; O'Hara *et al*., 2019, 2021). Furthermore, birds are not the only nest builders; all of the great apes build nests every night, both on the ground and arboreally, some of which involve complex construction techniques, such as bending and weaving branches (Anderson *et al*., 2019).

Although Hunt *et al*. (2013) disagreed with Hansell & Ruxton's (2008) views on tool use, they do share the idea that there is an essential difference between the tool use of birds and primates. Hunt *et al*. (2013) proposed a ‘bird model’ of flexible tool use, in which tool‐using birds like New Caledonian crows (*Corvus moneduloides*) and woodpecker finches have an ‘inherited disposition for a specific type of tool use’ (stick‐like tools) with limited social learning facilitating vertical transmission in the family unit (p. 111). This stands in contrast to the ‘primate model’, which relies on cognitive abilities such as conceptual knowledge about objects' use as tools, and both vertical and horizontal social learning and transmission (Hunt *et al*., 2013). Although this argument allows for a more nuanced approach to the role of evolution in tool use, we will argue that, especially in light of new evidence, this primate–bird divide is no longer consistent with the available data.

## III. INHERITED TOOL USE SPECIALISATIONS

### (1) The emergence of inherited tool use

The role of inheritance in tool use, we contend, is more nuanced than is usually considered. However, there is an important distinction to be made between an inherited behaviour that involves tool use, and one that can *facilitate* tool use. We refer to the latter as a *behavioural predisposition*, an inherited tendency to engage in a certain behaviour, which can be responsible for the onset of many forms of tool use but does not guarantee its emergence. When a species has a predisposition from an ecological specialisation that incorporates object‐related behaviours that resembles a specific type of tool use, it may facilitate the emergence of the latter. If individuals that perform this behaviour have a survival advantage, its frequency should increase through natural selection, so that tool using becomes an inheritable trait [a phenotypic evolutionary process, as suggested by Hunt *et al*. (2013)]. Thereafter, morphological or other behavioural changes to enhance tool using may also evolve, a process known as *secondary adaptation* (Gould & Vrba, 1982). Inheritance should not be taken to be synonymous with inflexibility, however. As we will discuss, a high degree of flexibility can be present in animals in which tool use has become an inherited species‐typical action pattern. Tool use that has evolved in this manner within a species can typically be identified by its intraspecies prevalence (but not necessarily ubiquity) and its resemblance to the ecological specialisation from which it derives, having become itself a specialisation (hereafter, referred to as an *inherited tool use specialisation*). It is important to emphasise that tool use that evolves in this way is not necessarily ‘stereotyped’ tool use, however, in the original sense of being rigid, uniform, and unvarying.

However, not all behavioural predispositions lead to such an evolutionary process. A species may have a behavioural predisposition for a specialised behaviour that facilitates the emergence of tool use without it becoming an inherited trait. This is because it may provide a survival advantage in one environment, but not another, and consequently tool use is not always present in a population that has that behavioural predisposition. In such a case, tool use is only limited to certain individuals, groups and/or contexts, but it still bears a resemblance to the original specialised behaviour that facilitated its onset. Such tool use is not necessarily more flexible than what emerges in the aforementioned tool‐use specialised species. Moreover, as we will discuss, species that are predisposed to more generalised behaviours may also develop tool use that is accordingly more generalised in form. Although these species may develop more diverse forms of tool use, once again this does not imply that such tool use is more sophisticated than those emerging from specialisations. And, as we will argue later, regardless of how general or specialised, we suggest all tool‐using species share one important behavioural predisposition: an intrinsically motivated predisposition to make object combinations.

### (2) The example of island birds

The standard approach for determining whether tool use has become an inherited specialisation, as opposed to being spontaneously innovated or socially acquired, is to see whether it emerges at a specific point during ontogeny in a species, given an appropriate environment, materials, and opportunities, even when isolated from conspecifics. For example, Hawaiian ‘alalā crows (*Corvus hawaiiensis*), New Caledonian crows, and Galápagos woodpecker finches that are raised in the absence of a tool‐using adult develop basic stick tool use (Kenward *et al*., 2005, 2006; Rutz *et al*., 2016a; Tebbich *et al*., 2001). In tool‐using corvids, such as New Caledonian crows, a likely facilitator for tool use has been suggested to be a predisposition for inserting objects into cavities from a behavioural adaptation for food caching, which is an ancestral trait to the family [Kenward *et al*., 2005, 2006, 2011; but see Hunt, Lambert & Gray (2007) and Hunt & Gray (2007), who suggest stick nest building as a potential origin]. This may also apply to the ‘alalā crows, whose behavioural repertoire before their extinction in the wild included frequently caching inside the crotches of trees (Sakai, Ralph & Jenkins, 1986). Woodpecker finches also forage in trees, with common behaviours including the removal of bark and the probing of crevices, moss, and leaves (Tebbich *et al*., 2002). Interestingly, the ’alalā crows were described as engaging in similar behaviours when foraging for invertebrates in a ‘woodpecker‐fashion’ in tree bark (Sakai *et al*., 1986, p. 213). Notably, some of the tools made by ’alalā crows in captivity have been made out of bark (Klump *et al*., 2018). Although we can never know for certain the precise behavioural predispositions that led to the evolution of such tool use, as all of these species have a history of extracting hidden food sources from trees, this would be a plausible first step towards probing with already familiar tree‐derived materials.

It seems unlikely to be coincidental that all three of these species are found on islands, where their ancestors likely faced less competition for embedded prey and fewer predators (Rutz *et al*., 2016a). Islands are well known for their unusual selection pressures, which can, for example, enable niche expansion through reduced interspecific competition, or force behavioural flexibility due to the inability to move away from environmental changes or deterioration (Sayol *et al*., 2018). In the dry forest of Gouaro‐Déva in New Caledonia, tool‐obtained beetle larvae contribute substantially to the crows' overall lipid intake and are important for meeting their daily energetic requirements (Rutz *et al*., 2010). Unsurprisingly with such benefits, there appears to have been selection for secondary morphological adaptations that enhance their tool‐using abilities, such as greater binocular overlap and shorter, straighter bills compared to most other corvid species (Matsui *et al*., 2016; Troscianko *et al*., 2012). In a probable case of parallel evolution, the ‘alalā crows appear to possess similar features (Rutz *et al*., 2016a). They likely derived considerable nutritional benefits as well, considering that when still living in the wild, they were observed to have spent the majority of their feeding activity in trees, presumably foraging on invertebrates (Sakai *et al*., 1986). Similarly, although it takes longer for woodpecker finches to extract prey with tools, food that is only accessible through tool use is particularly rich in proteins and fat, increasing foraging profitability when prey are more scarce (Tebbich *et al*., 2002). This is illustrated by the extreme prevalence of tool use in nearly all adults in the arid zone of Santa Cruz Island of the Galápagos during dry periods, where half of their prey is captured using tools. In comparison, tool use is far rarer in the evergreen, lush, wet cloud forest, where surface prey is more abundant (Tebbich *et al*., 2002).

#### (a) The flexibility within inherited tool use specialisations

Although basic stick tool use appears to be species‐wide in New Caledonian crows, the same is likely not the case with some of the more complex ‘pandanus’ stepped tools used in some regions, which are cut in one to four steps from the leaves of *Pandanus* spp., resulting in tapered tools that are both sturdy and pointed, with rigid barbs facing away from the narrow end (Hunt & Gray, 2003). Tellingly, captive adults that are naïve to making such tools do not manufacture them when provided with pandanus leaves, despite already being proficient stick tool users (Hunt *et al*., 2007). Along with evidence that juveniles who develop pandanus tool use have at least one pandanus tool‐using parent, it has been suggested that pandanus tool manufacture may need to be learned (Holzhaider, Hunt & Gray, 2010; Hunt *et al*., 2007). In fact, juvenile New Caledonian crows spend a lot of time closely watching their parents using tools, and do not reach adult levels of proficiency in pandanus tool manufacture for a year or longer (Holzhaider *et al*., 2010). It has been argued that social learning in New Caledonian crows may underlie the localised distribution of different tool types, in what some researchers have described as cultural transmission [Holzhaider *et al*., 2010; Hunt & Gray, 2003; but for alternative explanations see Jelbert *et al*. (2018) and Klump *et al*. (2015a)].

The extent to which learning and flexibility interacts with the natural tool‐using ability of New Caledonian crows is especially apparent in experimental work conducted in the laboratory or temporary catch‐and‐release aviaries. For example, there is research showing that they take into account the length and diameter of tools, can use multiple tools in a sequence, and select as well as safekeep appropriate tools for future use (Boeckle *et al*., 2020; Chappell & Kacelnik, 2002, 2004; Gruber *et al*., 2019; Klump *et al*., 2015a,b; Klump, St Clair & Rutz, 2021; Knaebe *et al*., 2017; Taylor *et al*., 2009, 2010; Wimpenny *et al*., 2009). The textbook example of New Caledonian crow tool excellence comes from the captive female ‘Betty’, who spontaneously innovated the manufacture of a hook tool from wire, in order to solve a tube task when she was unable to access a pre‐made tool (Weir, Chappell & Kacelnik, 2002). As New Caledonian crows have more recently been found to put a bend into the handle of hooked stick tools in the wild (Klump *et al*., 2015a), it has been argued that Betty's behaviour may have stemmed from an innate tool manipulation routine that does not require exceptional cognitive abilities (Rutz *et al*., 2016b). However, in that case, Betty would still be innovative in applying a pre‐existing behavioural routine to a completely new material in a novel functional context (e.g. a hook, not a handle). Tool bending also does not seem to be a ubiquitous behaviour in wild hook‐making New Caledonian crows (Klump *et al*., 2015a), and hooked‐stick tools seem to be limited to specific populations with high individual variation (Hunt & Gray, 2007).

Learning also plays a role in the tool use of other birds. Preliminary research on ’alalā crows, has shown that younger crows lack the dexterity of older crows, indicating a refinement of technique with practice (Rutz *et al*., 2016a). Despite Hall's (1963) argument that because the tool use of woodpecker finches is inherited, it must involve little learning, it was revealed that woodpecker finches can take advantage of a wide variety of materials as tools, to which they adjust their technique and movements (Millikan & Bowman, 1967). In an experiment by Tebbich *et al*. (2001), five juvenile woodpecker finches developed a novel upward levering technique used in an artificial horizontal tree trunk cavity that would not be functional in the wild. Clearly, having an inherited tool use specialisation does not necessarily mean the behaviour is rigid and lacking in variation (Kenward *et al*., 2006; Tebbich, Sterelny & Teschke, 2010). Depending on the cognitive abilities of the animal, a considerable degree of flexibility and innovation may be possible for such tool users.

### (3) Inherited components in human tool use

Further, the idea that inheritance is synonymous with inflexible, stereotyped tool use is completely undermined by the fact that the most flexible and innovative of all tool users are humans, and yet, at the same time, humans are arguably one of the most tool‐adapted animals. In comparison to other primates, there are a number of unique morphological features of the human hand, such as differences in thumb‐to‐finger length ratio, thumb muscle architecture, and radiocarpal wrist extension, which have been argued to represent secondary adaptations specifically to stone tool use. These include enabling forceful precision grips, cupping the hand to various object shapes, and stabilisation from the stresses of percussive actions (Marzke, 2013).

Moreover, Lockman (2000) has argued that the origins of human tool use can be found in the perception–action routines that infants display in the first year of life. Certainly, there appear to be rudimentary tool‐using behaviours early in human ontogeny that suggest a possible specialisation for percussive tool use as an inherited action pattern. For example, starting from approximately 6 months of age, children begin to hammer with objects, steadily transitioning into percussive tool use actions, as their movements become more regular, controlled, and aimed (Kahrs, Jung & Lockman, 2013a). From 2 to 3 years of age, children increasingly rely on their wrist while hammering, further improving their accuracy (Kahrs, Jung & Lockman, 2013b). The use of the wrist in percussion is mostly limited to humans within primates, and allows for increased precision during complex tool actions, such as stone knapping (Kahrs *et al*., 2013b). One language‐trained bonobo, Kanzi, learned to make stone flakes, but showed limited skill even after years of practice, most likely due to biomechanical constraints such as wrist morphology (Kahrs *et al*., 2013b; Schick *et al*., 1999). This stands in contrast to the performance of human participants, naïve to stone tool‐making techniques, who were able to innovate early *Homo* stone‐knapping techniques within a single session of a study (Snyder, Reeves & Tennie, 2022). It is unlikely, then, that the presence or absence of inherited tool use specialisations can be said uniquely to differentiate the tool use of primates and birds.

## IV. PREDISPOSITIONS AS FACILITATORS OF TOOL USE INNOVATIONS

### (1) Behavioural predispositions and the example of pounding in primates

If percussive tool use in humans has, in fact, become an inherited specialisation, its origin is likely in the behavioural predisposition to percuss open enclosed food items, such as shellfish, fruit and nuts, which is common among extractively foraging primates. However, as discussed, a behavioural predisposition does not inevitably result in tool use becoming an inherited behaviour. Still, having an ecological specialisation for percussing edible objects does appear to facilitate the emergence of percussing of non‐edible objects against a target (that is, pounding tool use). It is not surprising, then, that the predominant form of tool use in several species of capuchin monkeys (*Cebinae* spp.) and long‐tailed macaques (*Macaca fascicularis*) involves pounding tool use (Barrett *et al*., 2018; Canale *et al*., 2009; de A. Moura & Lee, 2004; Ferreira *et al*., 2009; Fragaszy *et al*., 2004; Luncz *et al*., 2017; Malaivijitnond *et al*., 2007; Monteza‐Moreno *et al*., 2020; Ottoni & Mannu, 2001; Tan, 2017; Waga *et al*., 2006). Notably, compared to other related non‐tool‐using macaque species, Balinese long‐tailed macaques have been found to engage in a greater variety of percussive behaviours with stones, which were also more frequent, enduring, and widespread than the percussive stone handling of Japanese macaques (Pelletier *et al*., 2017). Chimpanzees also engage in nut‐cracking with a tool, and sometimes also in conjunction with an anvil, but this behaviour appears to have a sensitive period for its acquisition and depends on a high degree of social learning, as it is not easily innovated (Koops *et al*., 2022).

Although pounding tool use is not at all ubiquitous in any primate species (except perhaps humans), it is still possible that such a predisposition could eventually lead to tool use becoming an inherited specialisation over evolutionary time, as seen with some of the island birds. It is certainly worthwhile to note that stone tool‐using long‐tailed macaques have only been found on islands in Thailand [Yao Noi Island (Luncz *et al*., 2017); Piak Nam Yai Island (Malaivijitnond *et al*., 2007); Koh Ped/‘Monkey’ Island (Muhammad *et al*., 2024); Koram Island (Tan, 2017); see also Cenni *et al*. (2024) for stone tool use artificially induced in long‐tailed macaques in a provisioned sanctuary on the island of Bali]. Remarkably, although most observed tool use thus far has been limited to bearded capuchins (*Sapajus libidinosus*), the only known groups of white‐faced capuchins (*Cebus capucinus imitator*) that engage in stone pounding live on islands in Panama [Jicarón Island (Barrett *et al*., 2018); Coiba Island (Monteza‐Moreno *et al*., 2020)]. The dietary benefits of tool use to open palm nuts have been shown to be substantial, with a gain of 46% in caloric intake found in bearded capuchins (Izar *et al*., 2022). Although these capuchins engage in tool use opportunistically, clearly access to such a rich source of energy could provide a survival advantage in a demanding environment. It has been suggested this is at least one contributing factor to why tool use is more prevalent in groups of *Sapajus* spp. capuchins in the Caatinga dry forest, where there is erratic rainfall and an extensive dry season that can lead to energy bottlenecks (de A. Moura & Lee, 2004). This is supported by the finding that, in the drier habitats of the Caatinga, active tool use sites doubled and tool use increased during dry periods (Emidio & Ferreira, 2012).

### (2) Primate tool innovations in the absence of a clear predisposition

At lower rates, both capuchin monkeys and long‐tailed macaques can innovate other forms of tool use that do not appear to have directly derived from a percussive behavioural predisposition. In the Caatinga, a number of groups of bearded capuchins use stones not only for pounding, but for digging and hoeing soil to access roots, tubers, arthropods, and insects (de A. Moura & Lee, 2004; Falótico, Siqueira & Ottoni, 2017; Mannu & Ottoni, 2009). Some groups in the area will also use sticks to probe for prey and dip for honey, which is also sometimes used in conjunction with pounding tool use to gain access to the probing site (de A. Moura & Lee, 2004; Falótico & Ottoni, 2014; Mannu & Ottoni, 2009). Additionally, one group of blonde capuchins (*Cebus flavius*) has been observed to use sticks to perforate termite nests and fish with them (Souto *et al*., 2011). Provisioned temple‐living, long‐tailed macaques in Thailand have innovated the use of human hair as dental floss (Watanabe, Urasopon & Malaivijitnond, 2007), and another similar group on the Indonesian island of Bali has developed tool use with a variety of objects for the likely purpose of more efficiently scooping water, as well as stone and stick tool use for masturbation (Cenni *et al*., 2020, 2022, 2023a; Cenni, Wandia & Leca, 2023b). Intriguingly, some of the aforementioned bearded capuchin groups also receive seasonal provisioning from humans (Falótico & Ottoni, 2014; Mannu & Ottoni, 2009). Provisioning has been suggested to promote behaviours that allow for the emergence of tool use, due to the relaxed selective pressures on foraging, at least in some cases (Cenni *et al*., 2020, 2022; Izar *et al*., 2022; Leca, Gunst & Huffman, 2008; Mannu & Ottoni, 2009; Waga *et al*., 2006; but for counterexamples see Muhammad *et al*., 2024; Spagnoletti, 2012).

Like provisioning, captivity can free up time for exploration, play, and close social contact, and thus can lead to more frequent and varied tool use than is observed in the wild (‘captivity bias’; Haslam, 2013). In sanctuaries and zoos, bonobos (*Pan paniscus*) show similar tool use diversity and complexity as chimpanzees (Gérard *et al*., 2022; Gruber, Clay & Zuberbühler, 2010). This stands in contrast to tool use in the wild, where, despite living in similar habitats with comparable tool use opportunities, bonobos do not use tools to the same extent as chimpanzees, especially not in a feeding context, as nearly all wild bonobo tool use that has been observed has been social or self‐directed in function (Furuichi *et al*., 2015; Samuni *et al*., 2022). Similarly, the few observations of individual gorillas (*Gorilla* spp.) using tools in the wild have also primarily been in a non‐foraging context (Breuer, Ndoundou‐Hockemba & Fishlock, 2005; Grueter *et al*., 2013; but for one foraging example see Kinani & Zimmerman, 2015), although in the case of gorillas, it has been argued that they do not need to feed using tools, as they do not rely on extractive foraging (van Schaik, Deaner & Merrill, 1999), and, when necessary, can mostly extract embedded food items using brute force (Fontaine, Moison & Wickings, 1996; Grueter *et al*., 2013; Parker & Gibson, 1977). Despite this, in a survey of a dozen zoos, nearly all of the gorillas were reported to use tools (Parker *et al*., 1999), and one study of captive gorillas showed that nearly all of the adults and adolescents had spontaneously become tool users and makers (Fontaine *et al*., 1996). There are also numerous reports of captive and rehabilitant orangutans (*Pongo* spp.) using tools in captivity (Lethmate, 1982; Russon & Galdikas, 1995; Shumaker, Walkup & Beck, 2011), and a number of zoos currently provide orangutans stick‐tool use opportunities as part of their enrichment (Forss *et al*., 2016). However, while some populations of Sumatran orangutans (*Pongo abelii*) habitually make and use a variety of tool types for extractive foraging in the wild, in Borneo such tool use is largely absent, despite Bornean orangutans (*Pongo pygmaeus*) having access to similar high‐value resources such as the *Neesia* fruit (Meulman & Van Schaik, 2013; van Schaik, Fox & Sitompul, 1996; Van Schaik & Knott, 2001). Importantly, when great apes are directly compared in experimental tool use studies, there are surprisingly few differences found between species (Girndt, Meier & Call, 2008; Herrmann, Wobber & Call, 2008; Manrique, Gross & Call, 2010; Manrique & Call, 2011; Martin‐Ordas, Call & Colmenares, 2008; Martin‐Ordas, Schumacher & Call, 2012; Mulcahy, Call & Dunbar, 2005; Mulcahy & Call, 2006a,b). The experimental support for the extensive captive tool‐using abilities in all of the great ape species strongly indicates that variability in the appearance of tool use in natural settings does not necessarily reflect actual tool use capacity, but rather that some tool innovations that lie within a species' ‘zone of latent solutions’ (i.e. within their capability to invent unaided; Tennie, Call & Tomasello, 2009) appear only in the right conditions.

In wild chimpanzees, there is incredible variation in the types of tool use exhibited by different communities, to such a degree that it has been argued that chimpanzees possess unique tool use cultures [in that specific tool use behaviours are socially transmitted and widespread but localised (Whiten *et al*., 1999); but see Schuppli & Van Schaik (2019) concerning how using localisation criteria can underestimate the extent of cultural repertoires]. For instance, whereas nut cracking has been found in some populations to the west of the Sassandra river in the Côte d'Ivoire, it is absent to the east, even in similar habitats with wide availability of both stones and nuts (Boesch *et al*., 1994). Furthermore, in East Africa, there appears to be no extractive stick tool use in the productive Budongo Forest, yet it is present in the less food‐stable Kibale, despite the two areas being in close geographic proximity (Gruber *et al*., 2012). Recently, an analysis of 144 chimpanzee communities indicated that greater environmental variability, especially distance from the more ecologically stable Pleistocene refugia, predicted behavioural diversity, including tool use (Kalan *et al*., 2020).

It has been a topic of great debate as to why tool use arises in some tool‐capable groups in the wild and not others [e.g. the ‘necessity hypothesis’, ‘opportunity hypothesis’, ‘relative profitability hypothesis’, ‘terrestriality hypothesis’, ‘limited invention hypothesis’, and ‘social tolerance hypothesis’ (Furuichi *et al*., 2015; Fox, Sitompul & van Schaik, 1999; Rutz *et al*., 2010; Rutz & St Clair, 2012; Gruber *et al*., 2010; Visalberghi *et al*., 2005; van Schaik *et al*., 1999)]. However, it should be noted that these are distinctly different from hypotheses concerning the internal processes leading to tool use; that is, they seek to explain *why* tool use appears in some groups, but not *how*. We suggest that the presence of high levels of tool use variation within a species indicates that, rather than having developed a particular specialisation resulting in only specific types of tool use, there is a more general ability to innovate a diverse range of tools. In tool‐using great ape communities, it has been estimated that the species‐wide repertoire in the wild for chimpanzees consists of 42 tool use variants, 38 for orangutans, and 13 for bonobos, with a wide range in the number employed by each community [6–22 for chimpanzees, 8–20 for orangutans, 8–10 for bonobos (Furuichi *et al*., 2015; Meulman & Van Schaik, 2013; Sanz & Morgan, 2007)].

### (3) Behavioural predispositions in non‐specialised tool‐using birds

Within birds, the expression of tool use may also be variable. There are several corvid species that are not recorded tool users in the wild but have shown a capacity to innovate tool use in captivity. Rooks (*Corvus frugilegus*) have been found to be able to use and manufacture tools on a variety of tasks (Bird & Emery, 2009), and one group of captive blue jays (*Cyanocitta cristata*) discovered how to rake pellets into their cage using torn pieces of paper (Jones & Kamil, 1973). There is also evidence for some captive tool use in ravens (*Corvus corax*; Jacobs & Osvath, 2023). The frequency of probing behaviour/stick tool use in these corvids suggests a similar origin to that of the New Caledonian crow, that is caching behaviour (Kenward *et al*., 2005, 2006, 2011).

Tool use may also emerge from behavioural predispositions in the wild but stay contained to certain populations. For example, male palm cockatoos (*Probosciger aterrimus*) drum with their feet against tree hollows in order to attract females. In Northern Australia, but apparently not New Guinea, the males create individually unique ‘drumsticks’ held in their feet to produce loud signature drumming (Heinsohn *et al*., 2017, 2023; note, however, that there has been less observational research effort in New Guinea). Possibly, Australian cockatoos started to incorporate the use of objects into their already established foot‐drumming routine, and this innovation spread through social transmission.

### (4) Flexible tool use innovations in parrots

Interestingly, for some cases of tool use, no behavioural predispositions have been identified. For example, unlike tool‐using corvids, parrots lack the predispositions that have been proposed to promote avian tool use, such as food caching (Kenward *et al*., 2005, 2006, 2011). Moreover, most parrots, as well as their ancestors, do not establish complex relations between sticks in order to weave nests (Eberhard, 1997, 1998). Despite having a beak morphology that makes handling sticks as tools incredibly challenging, kea parrots (*Nestor notabilis*) have been shown to innovate stick tool use successfully in experimental settings (Auersperg, Huber & Gajdon, 2011a; Auersperg *et al*., 2011b), and a captive kea innovated the use of a pebble to compensate for his missing upper mandible, in order to be able to preen himself (Bastos *et al*., 2021). There is also some evidence for keas using sticks to trigger stoat‐trap boxes in the wild (Goodman, Hayward & Hunt, 2018). Additionally, there are a number of anecdotal reports and pet videos of a wide variety of captive parrot species scratching themselves with a tool, although more experimental research is necessary to understand the phenomenon (Bastos *et al*., 2025; Boswall, 1977, 1978, 1983a,b). Such self‐care tool use should not be expected to arise frequently in the wild, as scratching in these cases appears to compensate for the lack of allopreening from conspecifics in captivity (Bastos *et al*., 2025).

The Goffin's cockatoo is likely one of the most proficient avian tool users. In captivity, some individuals have been able to innovate the use and manufacture of the same tool with different materials, as well as the manufacture of different tools with the same material (Auersperg *et al*., 2016; Laumer *et al*., 2017). They can also match tool length to distance, consider tool functionality, safekeep tools for later use, make prospective tool use decisions, socially acquire tool use, prosocially transfer tools according to a conspecific's need, and use composite tools as well as tool sets (Auersperg *et al*., 2012, 2014b, 2017, 2018; Beinhauer, Bugnyar & Auersperg, 2019; Laumer, Bugnyar & Auersperg, 2016; Osuna‐Mascaró *et al*., 2022, 2023). In their native habitat, the Tanimbar archipelago in Indonesia, some Goffin's cockatoos have been discovered to use and manufacture complex tool sets (O'Hara *et al*., 2021). There are also strong indications of feral Goffin's cockatoos using wood fragments as extractive tools in Singapore, where they have established a breeding population (Mioduszewska *et al*., 2023; Osuna‐Mascaró & Auersperg, 2018).

**Fig. 1:**
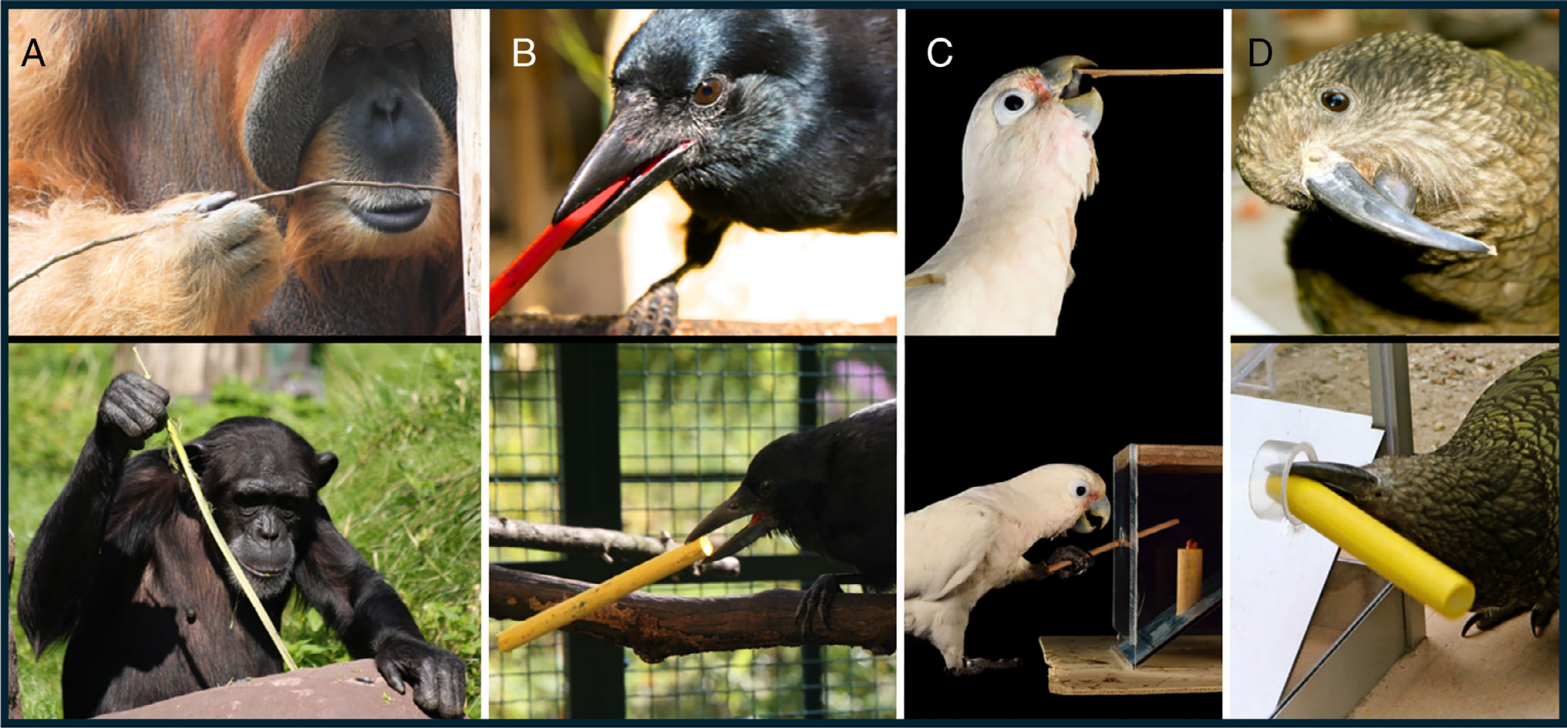
Morphological variation in tool‐handling ability. (A) Great apes' digital manipulation enables a high level of control, while being able to move the head for visual guidance. (B) The New Caledonian crow's secondary adaptations to tool use, i.e. increased visual overlap and a straight beak, enable precise control of a tool. (C) The Goffin's cockatoo's beak is adapted to seed de‐husking, thereby requiring repurposing to hold the tool by pressing the tongue against the upper beak tip, or alternatively by gripping the tool with its zygodactyl feet. (D) The kea's beak is more strongly adapted to digging and shovelling during extractive foraging, and holding a stick tool straight in the beak is morphologically impossible for this species, so that insertion of a stick can only be achieved by proximal handling and fiddling through the opening.

The Goffin's cockatoos' beak is not ideal for tool using, but they succeed in achieving a firm grip on their tools by repurposing their beak and tongue, which are shaped as an adaptation to seed de‐husking (Mioduszewska, Auersperg & O'Hara, 2022). While doing so, each individual appears to have their own specific technique, including holding the stick at the proximal or distal end during insertion, holding the stick between their mandibles or between their tongue and the upper mandible, or even using a foot instead of the beak to grasp the tool (Fig. 1) (Auersperg *et al*., 2014b; Osuna‐Mascaró *et al*., 2022). Such variability in technique, lack of secondary adaptations, and restriction of tool use to only some individuals, strongly indicates that their tool use is not an inherited specialisation, running counter to the suggestion that avian tool use must have a component of inheritance (Hunt *et al*., 2013).

As the examples we have described thus far show, most tool‐using species do not fit neatly into strict categories: a species may be a tool use specialist, and even possess specific secondary adaptations for tools, and yet exhibit a high degree of flexibility, whereas in other species, behavioural predispositions, which are themselves inherited specialisations, may facilitate specific forms of tool use to emerge with great variance in complexity. Finally, some types of tool use seem to lack any specific type of behavioural predisposition altogether.

## V. INTRINSIC MOTIVATION FOR OBJECT COMBINATIONS

### (1) Object combinations as a predictor of tool use

As we have seen, tool use emerges and develops in myriad ways and forms in animals. However, what is shared among all of these species is the pivotal role of object manipulation. Objects may be manipulated alone and/or non‐specifically (primary manipulation) or in relation to other objects or environmental features (secondary manipulation) (Torigoe, 1985); the latter requires considering objects and surfaces in reference to each other, and the complexity of these spatial relations increases the difficulty of their management (Fragaszy & Cummins‐Sebree, 2005).

The importance of object manipulation in the emergence of tool use has long been remarked upon historically (e.g. Alcock, 1972; Hall, 1963; Lancaster, 1968; Parker, 1974; Parker & Gibson, 1977; Schiller, 1957; Van Lawick‐Goodall, 1971). The extensive object manipulation in immature animals as a precursor to the emergence of tool use has been coined as the ‘preparation for tool use hypothesis’ (Koops *et al*., 2015b), which derives from the argument that one of the functions of object manipulation in the context of play is to enable young animals to practice their tool‐using skills (Smith, 1982). There have been several correlational studies explicitly linking object manipulation and tool use. For example, in a study spanning 74 species of primates, Torigoe (1985) found that the two groups that showed the most tool use also exhibited more complex object manipulation; more recently, Heldstab *et al*. (2016) obtained similar results in 36 species of primates. Noticeably, even among the great apes, despite all being competent tool users in experimental settings, chimpanzees show more diverse and higher levels of object manipulation than bonobos and gorillas, which are the same species that exhibit comparatively less tool use in the wild (Koops, Furuichi & Hashimoto, 2015a; Koops *et al*., 2015b; Lonsdorf *et al*., 2009; Ramsey & McGrew, 2005; but see Takeshita & Walraven, 1996). However, we contend that object manipulation more generally is not sufficient for the emergence of tool use, for one very important reason: there are many, many animals that manipulate objects, especially when building nests and processing or accessing food items, that never innovate tool use.

Alternatively, it has been suggested that a better predictor of tool use in animals is actually whether they engage in ‘generative’ types of object manipulation, which includes variations of actions towards one object, the same action applied to a variety of objects, and object combinations with other objects or surfaces (combinatory, or combinatorial, manipulation; Fragaszy & Adams‐Curtis, 1991). Put simply, the more frequently and variously an animal manipulates objects, the higher the probability that it will discover a potential use as a tool (Fragaszy & Adams‐Curtis, 1991). Tellingly, in the Torigoe (1985) study, it was specifically the tool‐using primate species that engaged in secondary object manipulation by physically combining objects with other objects or specific features in the environment. Torigoe (1985) made the argument that since tool use fundamentally relies on relating objects to other objects, such secondary manipulation was the most closely related to tool use. Thus, he suggested that tool use was more likely to arise from the extent to which objects were combined with each other, rather than more varied object manipulation alone (Torigoe, 1985). A focus on the relations between objects is now central to the action‐perception theoretical approach to tool use (Fragaszy & Cummins‐Sebree, 2005; Fragaszy & Mangalam, 2018; Lockman, 2000).

Following this perspective, the complexity of an animal's actions can be quantified by the underlying spatial relations, that is the consideration of objects, surfaces, and the body in relation to each other (Fragaszy & Cummins‐Sebree, 2005). Logically, the larger the number of relations to be detected and managed, the more difficult the task; additionally, complexity is argued to be affected by the specificity of these relations (the geometric alignment, placement and orientation), their temporal dynamics (static or dynamic) and order (static or concurrent), as well as the actor's frame of reference (egocentric or allocentric) (Fragaszy & Cummins‐Sebree, 2005; Fragaszy & Mangalam, 2018). Therefore, it follows that the most common combinatorial actions in capuchin monkeys consist of relatively simple single non‐specific relations between an object and a substrate (e.g. rubbing or pounding an object against the ground), while they rarely combine two objects together, which entails greater specificity (e.g. banging nuts against each other) (Fragaszy & Cummins‐Sebree, 2005). It also explains why egocentric tool use (the tool directed against one's own body, e.g. for scratching) is more widespread than allocentric tool use (directing a tool against another object/substrate), at least in parrots (Bastos *et al*., 2025) and non‐primate mammals (Colbourne *et al*., 2021), as the relation between self and object is simpler to manage than between two objects (Fragaszy & Mangalam, 2018). Fragaszy & Mangalam (2018) applied this model of spatial reasoning to their ‘tooling’ framework, a radical, action‐perception based re‐conceptualisation of tool use. Tooling focuses on the transformation of an actor grasping a tool into a ‘body‐plus‐object system’, and the actor's management of the spatial relations involved in creating a mechanical interface between the tool and target. As tool use is essentially a special form of combinatory object manipulation, the complexity of spatial relations also determines the complexity of tooling (Fragaszy & Mangalam, 2018). Furthermore, as tooling requires the creation of a deliberate mechanical effect, it inherently necessitates a certain level of specificity that can only be attained by species capable of making more spatially complex combinations.

This line of reasoning also leads to the prediction that not only will species that make more frequent combinations be more likely to exhibit tool use, but also that they will exhibit more spatially complex types of combinatory behaviour. In two related comparative studies on (extrinsically) unrewarded object combinations, which included nine species of parrots and three species of corvids, groups of conspecifics were given four activity plates made up of vertical tubes, horizontal tubes, shallow holes, and poles, alongside a variety of differently sized and coloured wooden cubes, balls, rings, and sticks (Auersperg *et al*., 2015, 2014a). The species that were known to be able to engage in tool use (Goffin's cockatoos, palm cockatoos, kea, and juvenile New Caledonian crows) combined free objects together at high rates and made specific combinations between objects and features of the activity plates (e.g. inserting objects into holes and tubes; stacking rings onto poles and tubes). Other species known for technical problem solving exhibited some combinatory behaviours [ravens, juvenile jackdaws (*Coloeus monedula*), and grey parrots (*Psittacus erithacus*)], but more limited in form compared to the tool users. Notably, it was only the two species known for the most complex and varied tool use in the wild and captivity, the Goffin's cockatoos and juvenile New Caledonian crows, that combined three free objects (‘triadic combinations’) (Auersperg *et al*., 2015, 2014a). Interestingly, despite the publication dates, the research by Auersperg *et al*. (2014a) actually took place with the Goffin's cockatoos before tool use emerged in the same captive group (Auersperg *et al*., 2012).

If, as we will posit, object combinations lead to tool use, one would predict that combinatory behaviour emerges first in development. Although necessarily correlational in nature, there is some evidence that during ontogeny, combinatory object manipulation emerges before tool use in numerous species [tufted capuchins, *Sapajus apella* (Fragaszy & Adams‐Curtis, 1991); chimpanzees (Hirata & Celli, 2003); New Caledonian crows (Kenward *et al*., 2005, 2006); orangutans (Schuppli *et al*., 2021); long‐tailed macaques (Tan, 2017); baboons, *Papio cynocephalus anubis* (Westergaard, 1993)]. A recent study adapted the activity plates used by Auersperg *et al*. (2014a, 2015) for children aged 15–24 months found that all ages engaged in similar levels of simple (less specific and/or static relations) and complex (more specific and/or dynamic) object combinations, whereas tool use performance increased with age (Rat‐Fischer *et al*., 2024). They also found that, within participants, children that exhibited higher levels of object combinations were also more successful at means–ends problem solving, including tool use tasks. Although research into the connection between tool use and object combinations specifically is still preliminary, we believe the work so far is compelling and will hopefully inspire further research both within and among species.

The study of object combinations in play and/or exploration may be especially fruitful, as combinatory behaviours appear to occur often in these contexts. By definition, object exploration suggests the goal of information‐seeking, whereas play has limited immediate functions (sometimes described as purposelessness; Burghardt, 2005). However, it can be challenging to distinguish between the two psychological states, as they are inherently intertwined: play may incorporate explorative elements (Pellis & Burghardt, 2017), and object exploration easily transitions into play and *vice versa* (Weisler & McCall, 1976). For our combinatory tool use origins hypothesis, both types of combinatory object manipulation are relevant. Importantly, neither object exploration nor play results in a direct extrinsic reward; it is the behaviour itself that is intrinsically motivating. Crucially, play and exploration with objects allows the animal to expand its behavioural repertoire by trying new combinations, producing flexibility that can lead to the emergence of different variants of tool use (Bruner, 1972). Such flexibility is one of the factors that Leca & Gunst (2023) have argued makes object play particularly suited as an exaptation for tool use.

### (2) Intrinsic motivation to combine objects

In addition to engaging in object combinations in the absence of an extrinsic reward, we suggest that the intrinsically rewarding nature of combinatory object manipulation is apparent in the repetition of certain actions with objects, akin to the ‘circular reactions’ described by Piaget (1952) in human sensorimotor development, in which infants repeatedly reproduce interesting effects that they create in their environment. In the more advanced tertiary stage of these circular reactions, the infant varies these repeated manipulations to see the changes in outcome as a form of ‘active experimentation’ (Piaget, 1952). Thus, an animal creates and observes the effects of repeating and varying its combinatory manipulation in a non‐stereotypic fashion [see Burghardt (2005) for important distinctions between playful and stereotypic repetition]. Like play, it is the process itself that is rewarding for its own sake (i.e. autotelic), rather than any specific end (Burghardt, 2005). Under low‐cost conditions, it has been suggested that it is pleasurable feedback that allows such behaviour to be maintained proximately (Leca & Gunst, 2023), and why the behaviour occurs in the absence of any direct extrinsic reward.

This is not to say that engaging in object combination does not have ultimate beneficial effects. In addition to the potential for innovation, it stands to reason that tool‐using animals with increased experience with objects can improve their related motor skills, as well as learn object affordances and the potential relations between different objects and surfaces (Lockman, 2000). It is probable, then, that tool‐using animals that gain more experience in this way are more likely to enhance their survival and reproduction, especially species with a tool‐use specialisation that depends on their tool use skills. This has led several authors to suggest that there may be evolutionary pressure in tool‐using species on their motivation generally to interact with objects (Alcock, 1972; Call, 2013; Hunt *et al*., 2013; Kacelnik, 2009; Kenward *et al*., 2006; Rutz *et al*., 2016a). However, as has been suggested for object play, it is also possible that such motivation for object interaction is an evolutionary by‐product, rather than specifically selected for, but the benefits are such that it has been maintained and not been selected against (Leca & Gunst, 2023).

There is some evidence showing variation in the motivation to combine objects in species that should be equally capable of doing so, from a morphological and motor standpoint. In bonobos, it has been argued that there may have been a trade‐off between the motivation for general object interaction and social attention, resulting in lower rates of tool use compared to chimpanzees (Koops *et al*., 2015a). Immature and adult orangutans in a tool‐using area of Sumatra show greater exploratory object manipulation than Bornean orangutans that do not engage in complex extractive tool use (Schuppli *et al*., 2017). A study of ravens and New Caledonian crows found that, despite having similar caching‐related combinatory action patterns early in ontogeny, the New Caledonian crows continued to make functional combinations at high rates, even after they could successfully use tools, while the ravens' behaviour was replaced by actual caching (Kenward *et al*., 2011). Interestingly, a similar developmental decline in general object exploration occurs in carrion crows (*Corvus corone, C. cornix*), although the crows did not exhibit as high a frequency for object exploration as ravens during juvenility (Miller *et al*., 2015). In the case of New Caledonian crows, it could be argued that tool use developed first, and the motivation to combine objects is a by‐product or secondary adaptation for their tool‐using specialisation; however, in the great apes that lack such a specialisation, with individuals and groups that do not always innovate tool use, the parsimonious explanation is that the motivation to combine objects takes precedence. This is an area that is ripe for further research, but we hazard the prediction that individuals and species that show a greater motivation to combine objects will also have a higher probability of innovating tool use.

### (3) Tool use variation of specialists and opportunists

We also predict that the types of object combinations exhibited by a species are aligned with their ecological niche. It has been noted that several tool‐using animals, including humans, have tendencies to engage in species‐specific action patterns that affect the kind of tool use actions that may ultimately emerge (de Resende, Ottoni & Fragaszy, 2008; Fragaszy & Mangalam, 2018; Lockman, 2000). We have made the argument that, in some species, an inherited predisposition for another behaviour facilitates tool use, and we hypothesise the mechanism by which this happens is the intrinsic motivation to engage in object combinations related to this predisposition. It is already known that this occurs in play, which usually contains elements of species‐typical activities (Burghardt, 2005). In the case of inherited tool use, object combinations deriving from a tool use specialisation may also become play behaviours, as suggested to have occurred in juvenile New Caledonian crows (Kenward *et al*., 2011), although it is questionable how distinguishable these behaviours would be from the original behavioural predisposition that facilitated this specialisation in the first place. However, play is not a necessary component; as argued as to have occurred in the digger wasp, a drive to engage in a pre‐existing object‐related mechanical action can lead directly to the tool use behaviour [Hunt *et al*., 2013; but for a case of apparent object play in insects, see Galpayage Dona *et al*. (2022), which is suggested to improve bumblebees' (*Bombus terrestris*) motor skills for flower extraction]. An ‘object‐related mechanical action’ is essentially a behavioural predisposition for a specific combination, and in such a case leads to extremely inflexible tool use (hence the designation of ‘stereotyped’), but this is not necessarily the case for other species. Long‐tailed macaques, for example, may engage in types of combinatory behaviours related to their predisposition for percussion, and consequently be more likely to innovate related forms of tool use; this has recently been illustrated nicely in a study in which Balinese long‐tailed macaques that showed a high percussive stone‐handling profile had an increased likelihood of innovating stone tool use to solve a percussive puzzle box [notably, this was not the case for a non‐combinatory behaviour and task (dropping); Cenni *et al*., 2024]. Stone handling, a socially influenced type of object play in provisioned macaques, thought to be a by‐product from relaxed foraging pressure, has been suggested to be a particularly co‐optable ‘exaptive pool’ of object‐related behaviours, due to its structural and functional similarities to tool use (Leca & Gunst, 2023). Certainly, although long‐tailed macaques appear to develop percussive tool use more frequently, they are capable of innovating other forms of stone tool use, as well as non‐stone tool use, and can show a great degree of flexibility.

We hypothesise that it is very likely that long‐tailed macaques are capable of innovating other tool use forms because they are opportunistic extractive foragers, in addition to their percussive specialisation. The importance of being an extractive forager for the development of tool use was proposed by Parker & Gibson's (1977) extractive foraging hypothesis, which argued that the ability to apply general complex object manipulation schemata is the foundation of flexible tool use. They suggested that the ability for complex object manipulation was selected for in omnivorous primates that required access to higher energy embedded foods, requiring manual dexterity to exploit, which were unreliable, unexpected, and/or seasonally available. Taking into consideration that most primate and avian tool use occurs in the context of obtaining otherwise inaccessible food (Pal & Sinha, 2022; Parker, 2015), opportunistic extractive foraging is clearly important. Of course, not all extractive foragers are tool users (Gibson, 1986; Parker, 2015). We predict that in order to create the foundation for flexible tool use, opportunistic extractive foragers need to pass a threshold in the complexity of their object manipulation, wherein they start to move from general manipulation to active combinations of objects with other objects/substrates in diverse and investigative ways. Such species frequently rely on their physical problem‐solving abilities, and the benefits of exploring their environment and developing a variety of different skills make them adaptable to a wide range of environments. In such a case, their object combinations can be expected to be more diverse and less predictable compared to those of specialists, which are more likely to be motivated to combine in a way related to specific behavioural predispositions, possibly both as a side effect of those predispositions and as a way to hone their related skills.

**Fig. 2:**
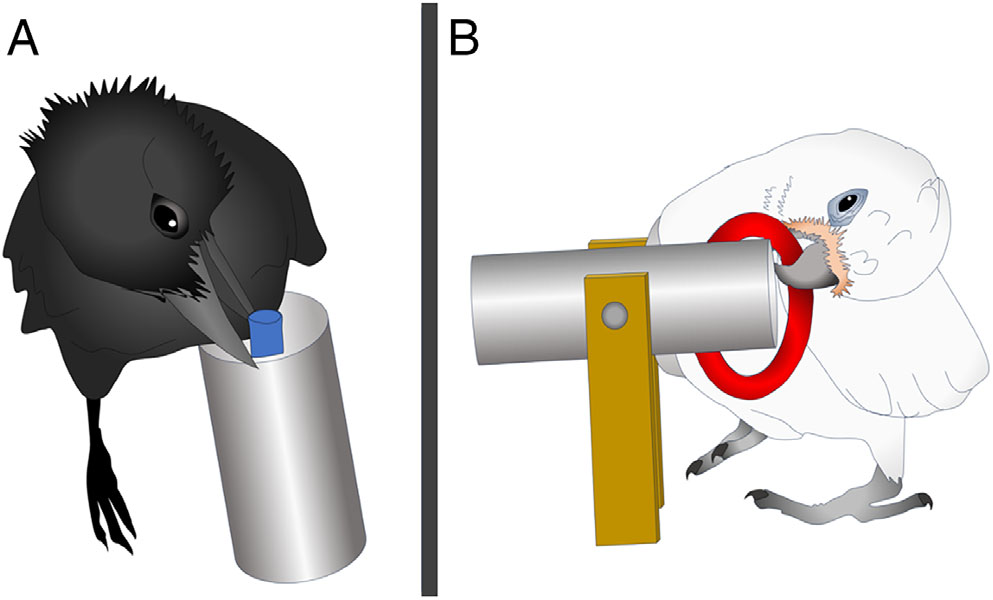
In Auersperg *et al*.'s (2015) extrinsically unrewarded playground experiment, it was found that (A) New Caledonian crows showed predominantly inserting‐type actions with non‐food objects at very high levels, whereas (B) the combinatory object actions exhibited by Goffin's cockatoos included both insertions and ring stacking.

The difference between the object combinations of specialists and opportunists is illustrated in the previously discussed work by Auersperg *et al*. (2014a, 2015) that directly compared the object play of several species of parrots and corvids. While it was only the Goffin's cockatoos and New Caledonian crows, the most tool‐advanced species, that exhibited triadic combinations, in addition to high rates of combinations of objects with other objects and activity plate features, the types of combinatory object manipulation that they expressed differed. Whereas the Goffin's cockatoos engaged in both inserting and ring stacking combinatory behaviours at the activity plates offered in the experiment, the New Caledonian crows mostly inserted and never stacked rings (as well as occasionally caching objects inside the aviary, which was much more common in the more caching‐adapted ravens) (Auersperg *et al*., 2015). Likely, the New Caledonian crows were expressing their intrinsic motivation to engage in insertion behaviours when combining objects, related to their tool use adaptation for probing with stick tools, while the Goffin's cockatoos exhibited more diverse combinatory manipulation, reflecting their opportunist–generalist extractive foraging lifestyle (O'Hara *et al*., 2019) (see Fig. 2). Notably, wild Goffin's cockatoos have been observed to make extrinsically unrewarded combinations, even during extractive foraging, suggesting an intrinsic motivation to combine: in fact, both tool‐using and non‐tool‐using birds in a capture‐release aviary were observed combining leaves, wood and other objects while actively feeding on fruit, and feral Goffin's cockatoos similarly combine vegetation with food (O'Hara *et al*., 2021; Osuna‐Mascaró & Auersperg, 2018).

### (4) The rarity of tool use and exceptions that support the rule

What is interesting is when an animal appears to lack a strong intrinsic motivation to combine. There are innumerable species that have the necessary morphology, dexterity and manipulative ability to use tools. In the laboratory, a number of animals have been explicitly shaped and trained to use tools with hundreds or even thousands of repeated trials and sequential steps, such as Japanese macaques (*Macaca fuscata*; Ishibashi, Hihara & Iriki, 2000), common marmosets (*Callithrix jacchus*; Yamazaki *et al*., 2011), large‐billed crows (*Corvus macrorhynchos*; Kanai *et al*., 2014), common crows (*Corvus brachyrhynchos*; Powell & Kelly, 1975), and common degus (*Octodon degus*; Okanoya *et al*., 2008). Yet, none of these animals, and countless other equally capable species, is known habitually to use tools in the wild thus far.

One explanation for the rarity of tool use in the wild is that tools are seldom more useful than anatomical adaptations (referred to as the ‘lack‐of‐utility hypothesis’; Hansell & Ruxton, 2008). On the contrary, Hunt *et al*. (2013) contend that there are actually substantial ecological benefits to the exploitation of otherwise unattainable resources through the use of tools (termed the ‘excess‐of‐opportunity problem’), and that other factors contribute to its rarity, depending on the nature of the tool use. In the case of stereotyped tool use, Hunt *et al*. (2013) believe its phenotypic evolution depends on whether there is already a pre‐existing object‐related mechanical action, whereas the constraints for the emergence of flexible tool use are cognitive. While current evidence supports a relationship between more flexible forms of tool use and cognition, we argue that the primary predictor of the onset of tool use is the intrinsic motivation for extrinsically unrewarded object combinations. Essentially, animals that express diverse combinatory behaviours are more likely to innovate diverse tool use behaviours, whereas cognition is a mediating factor in its expression. Ultimately, we propose it is the motivation to combine that underlies the rarity of tool use: if an animal does not make many object‐related combinations as part of its behavioural repertoire, especially in a foraging context, tool use is unlikely to emerge naturally, lacking any basis to do so.

Although combining objects with other objects is not common, combining objects with the self is somewhat intrinsic to many animals (e.g. processing food, rubbing against objects to scratch, applying materials such as sand, mud or oil to the skin/feathers/fur). It is telling that, while tool use seldom occurs in non‐primate mammals, ‘scratching’ of the self is the most frequently observed sub‐mode of tool use (Colbourne *et al*., 2021). According to Shumaker *et al*. (2011), ‘anting’ behaviour in birds, in which ants are applied to the plumage for the purpose of feather maintenance, occurs in over 200 species across several avian orders. If anting is tool use, as Shumaker *et al*. (2011) consider it to be, then rather than being rare, tool use would actually be very common in birds. Similarly, a recent paper by Bastos *et al*. (2025) suggests that tool use is more widespread across Psittaciformes than previously thought, as self‐scratching has now been observed in 28 species of parrots kept as pets. Although tool use in the context of self‐care is an interesting phenomenon in its own right, it is notable that a behaviour like ‘anting’ is overlooked as ‘tool use’ in large‐scale phylogenetic analyses (e.g. Lefebvre *et al*., 2002; Iwaniuk *et al*., 2009; Overington *et al*., 2009) and absent in the debate over the rarity of tool use (e.g. Hansell & Ruxton, 2008; Hunt *et al*., 2013), despite technically meeting the criteria of a wide definition such as that of Shumaker *et al*. (2011). We suggest this is because self‐directed, egocentric tool use is a fundamentally different phenomenon than allocentric tool use (and a simpler one to innovate, when taking into consideration the spatial relations to be managed, as posited by Fragaszy & Managalam, 2018). It is therefore important to distinguish between an intrinsic motivation to combine objects with the self from a motivation to combine objects with other objects/surfaces in the environment, as one is part of the normal behavioural repertoire of a multitude of species, whereas the other is more complex and seems to be extremely rare. In our own hypothesis presented here, we are referring to the onset of allocentric tool use. Egocentric tool use merits its own discussion.

## VI. COMBINATORY TOOL USE ORIGINS HYPOTHESIS

### (1) The onset of tool use as a spectrum

If all allocentric tool use, regardless of whether it is an inherited specialisation or a spontaneous innovation, derives from combinatory object manipulation, it may be a more unitary phenomenon than has previously been suggested. The current weight of evidence does not support a clear division of tool use across species lines, including between birds and primates. Fundamentally, we argue, it is the nature of the object combinations of a tool‐using animal that influences the features of the tool use that emerges. An opportunist–generalist extractive forager that relies on unpredictable and inaccessible resources will typically show a more diverse set of extrinsically unrewarded object combinations. The resulting tool use will consequently be less predictable, and a greater variety of tool types, modes, and techniques will likely result. Correspondingly, we propose that a more specialised animal that has an intrinsic drive to combine objects in a more specific manner, dictated by a pre‐existing ecological specialisation (that is, a behavioural predisposition), could express a narrower range of tool behaviour. Over evolutionary time, especially in the presence of strong selection pressures, this type of tool use is more likely to become an inherited tool use specialisation.

This, of course, does not mean that tool use that arises from a specialisation, whether it has become inherited or not, cannot be modified by mediating factors such as cognitive ability, manipulative skill, or the social/physical environment. As has been discussed, tool use that is specialised in origin can be expressed quite flexibly, and it would be misleading to try to create a sharp dichotomy between specialist and opportunist tool users. In fact, as we have seen, both specialised and generalised object combinations may appear in the same species, although in specialists the proportion of tool innovations may be skewed towards a specific tool type and tool use mode.

### (2) Tool use spectrum and previous research

**Fig. 3:**
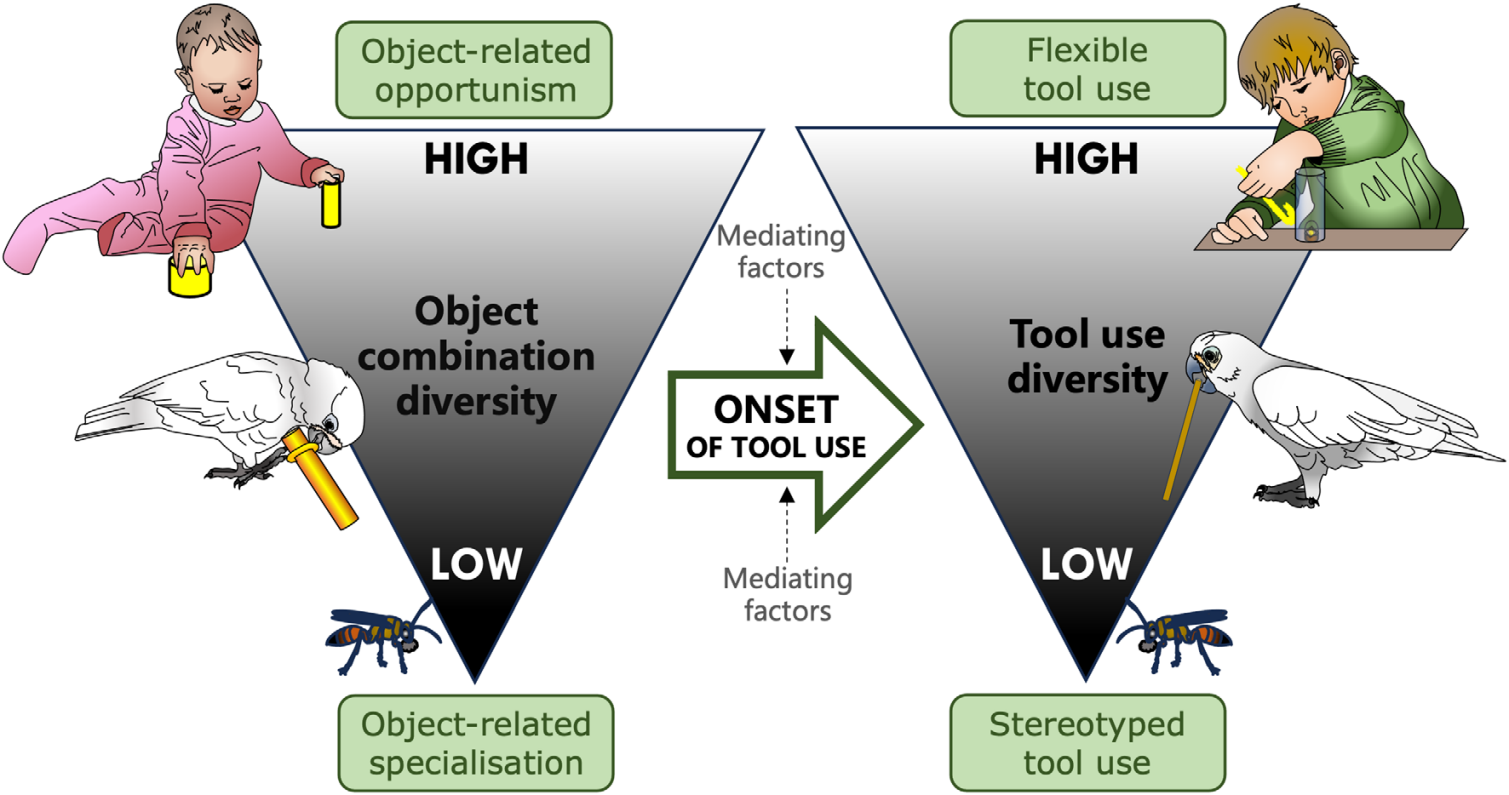
Diagram of the onset of tool use. The variety of combinatory actions an animal shows (combinatory spectrum; left) is predictive of the variety of tool use modes expressed by the species (tool use spectrum; right). The onset of each tool use mode is further acted on by mediating factors (e.g. cognitive abilities, morphology, motivation, resource availability) that affect the animal's ultimate position in the tool use spectrum.

We hypothesise, therefore, that the onset of animal tool use is based on a spectrum of combinatory variation (Fig. 3). We feel that this approach is compatible with, and supported by, most tool use work so far. Other researchers have remarked upon the importance of having an intrinsic motivation for more general object manipulation (Call, 2013; Hall, 1963), and many authors have also observed the importance of making object combinations to tool use (e.g. Auersperg *et al*., 2014a; Bruner, 1972; Fragaszy & Mangalam, 2018; Leca & Gunst, 2023; Lockman & Kahrs, 2017; Matsuzawa, 2001). Hunt *et al*. (2013) emphasised the importance of pre‐existing object‐related actions, and it has long been noted that species‐typical motor action patterns affect the discovery or development of tool use (de Resende *et al*., 2008; Fragaszy & Mangalam, 2018; Lancaster, 1968; Lockman, 2000; Schiller, 1957; van Lawick‐Goodall, 1971). Moreover, it is certainly possible that a number of the hypotheses that explain the presence or absence of tool use in already tool use‐capable species additionally contribute to its manifestation.

Our view also fits very well with the new tooling framework, which argues that it is through species‐typical exploratory actions with grasped objects that animals can learn the affordances related to tooling (Fragaszy & Mangalam, 2018). This is complementary to our contention that an intrinsic motivation for specialised combinatory actions driven by ecological adaptations can facilitate the emergence of specific types of tool use. The tooling framework also offers an explanation for the correlation between brain size and tool use; in order to tool, perceptuomotor complexity increases as the end‐effector shifts (‘distalises’) from the limb to the tool (Arbib *et al*., 2009), and the detection and management of spatial relations inherent in various forms of tooling relies on an inextricable link between perceptual motor pathways and cognitive skill (Fragaszy & Mangalam, 2018). As discussed, the complexity of these spatial relations has been argued to be affected by frame of reference, number of relations, specificity, temporal dynamics and temporal order (Fragaszy & Cummins‐Sebree, 2005; Fragaszy & Mangalam, 2018). Therefore, more complex tooling tasks require comparatively more cognitive power, and accordingly appear to be far rarer in animals than simpler tool use tasks (Colbourne *et al*., 2021). For example, to engage in a concurrent tooling task, in which two tools are used simultaneously, not only does the user need to consider the relation between the hand and tool, as well as the tool and target, but this management is doubled. It is possibly for this reason that children in one tool study were only able to innovate concurrent tool use at an older age, associated with a later stage of neurocognitive development, compared to their ability to solve the same tool use task in a sequence (Colbourne, Auersperg & Beck, 2024), and why there are no reported cases of animals using two tools at the same time [Colbourne *et al*., 2021; Shumaker *et al*., 2011; notably, Fragaszy & Mangalam (2018) predict such concurrent tooling is beyond the capacity of animals]. As opportunist extractive foragers naturally engage in a greater variety of behaviours that entail more diverse spatial relations of varying complexity, particularly those involving the combination of objects, it is perhaps not surprising that there is a relationship between brain size, extractive foraging and tool using (Navarrete *et al*., 2016; Parker, 2015; Reader *et al*., 2011). It is possible that cognitive ability plays less of a role for some specialised tool users, especially the more extreme ‘stereotyped’ animals, beyond what is strictly necessary to perform the tool behaviour (and its associated behavioural predisposition), but, as discussed, there can be great flexibility in specialised tool users that in some cases implies considerable cognitive abilities. In other words, brain size and other neural measures may not predict the onset of tool use (otherwise, one would expect tool use to be far more widespread, and especially to be present in more closely related species), but one should certainly expect a correlation. We hope to inspire further research into this possibility, for there is much still to be learned about the cognitive underpinnings of tooling.

### (3) Predictions from the combinatory tool use origins hypothesis

Accordingly, we believe that our proposal is compatible with, and well‐supported by, preceding research and evidence. Therefore, we can formally summarise our hypothesis as follows:

*As combinatory behaviour increases, so does the probability that (allocentric) tool use will be discovered. Furthermore, the type of tool use expressed has an increased probability of being similar in form to the type of combinatory behaviour expressed*.

We suggest that the close relationship between combinatory behaviour and tool use derives from tool use being a form of object combination in itself, but one that is extrinsically motivated and intentionally directed towards a more specific end. Thus, intrinsically motivated combinatory actions with objects provide the necessary scaffolding to enable the emergence of tool use. Combining objects with other objects or surfaces not only increases the probability of discovering tool use actions, but also developing the requisite sensorimotor skills and affordance learning for the skilful use of objects that may be used as tools.

The combinatory tool use origins hypothesis generates a set of five predictions that apply both within and among species.

(1) The main prediction is that species that are intrinsically more motivated to combine objects will be more likely to innovate tool use at higher rates.
(2) Tool‐using animals will exhibit relatively more spatially complex combinations (i.e. frame of reference, specificity, temporal dynamics, and order; Fragaszy & Cummins‐Sebree, 2005), especially in comparison with closely related non‐tool users.
(3) In species that exhibit an intrinsic motivation to combine objects, we expect that environments that enable increased rates of intense object exploration and play will subsequently also increase combinatory object actions and thereby tool use, i.e. captive or provisioned sites where foraging pressures are relaxed (e.g. Haslam, 2013), and/or geographically remote locations where predation is decreased (e.g. Rutz & St Clair, 2012).
(4) Similarly, we predict that the period of development with the highest rates of object combination should also be the time frame when tool use is most likely to emerge (albeit in a relatively rudimentary form), and that, ontogenetically, object combinations will precede tool use, since each is necessary for the other to emerge.
(5) Finally, and importantly, we predict that the expression of tool use behaviour should be congruent with the form of object combinations that are typically produced (e.g. insertion‐type combinations should lead to insertion‐type tool use), and that animals that show a wider variety of combinations will exhibit a wider variety of tool use. Thus, species that are specialised and/or possess specific behavioural predispositions should exhibit a narrower range of related types of tool use, whereas opportunist–generalists should show greater variation.

We suggest that the starting point to begin testing such predictions is by building ethogram‐style profiles of combinatory behaviour, which should aim to capture frequency, nature (e.g. inserting, percussing, rubbing), spatial relations (e.g. object–object, object–substrate) and level of complexity (following Fragaszy & Cummins‐Sebree, 2005). Moreover, they should also account for the developmental stage, environment (e.g. captive, provisioned, urban, etc.), and ecological niche (particularly foraging strategy, i.e. level of specialism/opportunism). The recent study by Cenni *et al*. (2024), using an action‐perception perspective, approximates such an approach. They recorded the stone‐handling behavioural patterns of juvenile, subadult, and adult Balinese long‐tailed macaques in a provisioned sanctuary in order to create stone‐handling profiles (dropping and percussive), which were then compared to their subsequent performance on stone tool innovation tasks (although note that they focused on general object play, rather than combinatory behaviour specifically). Other studies have also characterised specific types of combinatory behaviour and have linked these to existing tool use behaviour in groups of animals, such as the insertion behaviour of New Caledonian crows with their stick‐probing tool use (Auersperg *et al*., 2015; Kenward *et al*., 2006) or the predominance of object–substrate type combinations in captive capuchins with their nut‐cracking behaviour (Fragaszy & Adams‐Curtis, 1991), but the Cenni *et al*. (2024) study makes a direct link between the characteristics of the object actions made and the resulting innovative behaviour.

Creating profiles would also be useful for phylogenetic comparisons. There have been some endeavours to compare closely related tool‐using and non‐tool‐using species in terms of their combinatory behaviour, such as Kenward *et al*.'s (2011) comparison of the object combinations of New Caledonian crows and ravens during ontogeny, and Pelletier *et al*.'s (2017) study of the stone‐handling behaviour of Balinese long‐tailed macaques and two non‐tool‐using macaque species. Auersperg *et al*. (2014b, 2015) compared multiple species of parrots and corvids with respect to object combinations in play and tool use. There is also a study currently underway comparing object combinations in play, innovation and tool use behaviour in Goffin's cockatoos and two closely related corella species (J. A. D. Colbourne, M. O'Hara, Ö. Nasa, J. K. Colbourne, A. Wilkinson & A. M. I. Auersperg, in preparation). Making such direct juxtapositions, in which the same or similar methodologies and experimental settings are used to compare closely related species, is especially useful for pinpointing the differences between species that develop tool use and those that do not. We hope more studies of this kind will provide more evidence for the link between the motivation to combine objects and tool use. Such work will also provide data for large‐scale phylogenetic comparisons and meta‐analyses.

## VII. CONCLUSIONS

(1) In earlier tool use research, animal tool use was conceptualised as either operating on pure instinct with no learning and limited to a single context with little individual variation, or as requiring learning and/or innovating tool use flexibly in an ‘intelligent’ fashion. We highlight the difference between an inherited species‐wide tool use specialisation and a behavioural predisposition, an inherited tendency to engage in another behaviour, which can facilitate the emergence of tool use. Tool use specialisations may initially arise from a behavioural predisposition, but, through natural selection, spread throughout the population to become an inherited behaviour.
(2) However, as we have discussed, possessing such a specialisation does not necessarily result in inflexible, stereotyped tool use, nor does having a behavioural predisposition, although certain types of tool use related to that behavioural predisposition are more likely to arise. This is not only the case in birds, for many tool‐using primates have behavioural predispositions resulting in specific types of tool use. Meanwhile, there are bird species that do not appear to have any specific behavioural predisposition facilitating their tool use innovation. Therefore, taking into account the most recent evidence, it is difficult to claim that there are fundamental differences across taxonomic lines, including the previously proposed primate–bird divide.
(3) There are, nevertheless, clearly some species that develop more diverse types of tool use than others. Although it has been suggested that object manipulation may have a role in tool use, we are putting forth the hypothesis that the intrinsic motivation to combine objects in the absence of an external reward underlies the onset of tool use in all animals. Based on the evidence reviewed thus far, we propose that within these tool‐using animals, it is the nature of this combinatory behaviour that influences the tool use that emerges.
(4) Typically, opportunist–generalist extractive foragers will make more general combinations and show a greater variation of tool types, whereas specialists, which are more likely to have behavioural predispositions, will exhibit less‐diverse types and/or modes of tool use. At one extreme, this may result in a single type of tool use evolving that appears to be very rigid, or ‘stereotyped’. While this may be the case in a few species, other animals that have inherited tool use specialisations may still display a range of flexibility in their tool use expression depending on several mediating factors, such as their morphology and cognitive abilities. Even humans, despite being considered at the pinnacle of tool use flexibility, appear to possess strong secondary tool use adaptations and specialisations. Other species may be capable of innovating tool use, yet may only do so in particular environments.
(5) And so arises a very complicated picture, but all of these tool‐using animals still fall on the same rich spectrum of tool use, for they do share with each other one rather unusual quality: the motivation to combine.

## CONFLICTS OF INTEREST

None of the authors have a conflict of interest to disclose.

## Data Availability

Data sharing not applicable to this article as no datasets were generated or analysed during the current study.
